# Neutrophil dynamics in pulmonary fibrosis: pathophysiological and therapeutic perspectives

**DOI:** 10.1183/16000617.0139-2024

**Published:** 2024-11-27

**Authors:** Louise Elizabeth Crowley, Robert Andrew Stockley, David Richard Thickett, Davinder Dosanjh, Aaron Scott, Dhruv Parekh

**Affiliations:** 1 Birmingham Acute Care Research Group, School of Infection, Inflammation and Immunology, University of Birmingham, Birmingham, UK; 2 Respiratory Medicine, University Hospitals Birmingham NHS Foundation Trust, Birmingham, UK; 3 Birmingham Biomedical Research Centre, University Hospitals Birmingham NHS Foundation Trust, Birmingham, UK; 4 Joint senior authors

## Abstract

The shared pathobiological mechanisms driving progressive fibrosis in interstitial lung diseases (ILDs) remain unclear. Neutrophils, the most common immune cells in the human body, contain an extensive array of proteinases that are important for cell function, including tissue repair and remodelling. Increasing observational studies have reported elevated neutrophil counts in the respiratory tract and circulation of patients with ILD and suggest a role as a biomarker of disease severity. Neutrophils and their contents (including the formation of neutrophil extracellular traps (NETs)) are present in fibrotic lung tissue. Proteinases and NETs may drive fibrogenesis in animal and *in vitro* models and may impact transforming growth factor-β1 activation. However, the effect of neutrophil action, whether reparative or pathologically destructive to the delicate lung architecture, has yet to be determined. This review aims to summarise the current literature surrounding the potential role of the neutrophil as a biomarker and contributor to the pathogenesis of ILD. There is currently a paucity of treatment options in ILD driven by the knowledge gap underlying the overall disease mechanisms. This review concludes that neutrophils warrant further evaluation as manipulation of recruitment and function could provide a novel and much needed therapeutic strategy.

## Shareable abstract

**Proteinases and NETs are present in fibrotic lung tissue. Neutrophil elastase is implicated in collagen deposition and TGF-β activation. Neutrophils may be an overlooked marker for disease severity and avenue for therapeutic modulation in ILDs.** https://bit.ly/4gdNpEQ

## Introduction

Interstitial lung diseases (ILDs) are a group of nonmalignant lung parenchymal disorders encompassing a range of clinical phenotypes with varying degrees of fibrosis and/or inflammation. Idiopathic pulmonary fibrosis (IPF) is the most common subtype, characterised by increased collagen deposition, lung architectural destruction and breathlessness, with a median survival of ∼3 years if untreated [1]. Over a third of other fibrosing ILDs when progressive have a similar prognosis [2]. The collective term “progressive fibrotic interstitial lung diseases” (PF-ILDs), or newly defined “progressive pulmonary fibrosis” (PPF) by the latest American Thoracic Society (ATS)/European Respiratory Society/Japanese Respiratory Society/Latin American Thoracic Association clinical practice guideline [3], describes all fibrotic ILDs that demonstrate a progressive pattern based on fibrosis, symptoms, lung function and/or radiological features. The shared disease course and historadiological patterns seen in PF-ILDs support the notion that these conditions probably have at least some shared pathobiological mechanisms.

Neutrophils are the most common immune cell in the blood and one of the body's first responders to infection and tissue damage. Following extravasation and chemotaxis into target tissues, neutrophils can activate an array of inflammatory and antimicrobial responses [4]. These include phagocytosis, containment and destruction of organisms by proteinases and reactive oxygen species (ROS), and release of neutrophil extracellular traps (NETs). All of these processes have the ability not only to clear infection, but are critical in driving tissue injury and repair [5, 6].

Neutrophils have largely been neglected in the pathological paradigm of lung fibrosis. The clinical significance of increased neutrophils in the bronchoalveolar lavage fluid (BALF) of patients with ILD is questionable. Although neutrophils and their contents have been localised within fibrotic lung tissue [7], the temporal relationship of neutrophil mobilisation and disease pathogenesis is unknown. This raises the important issue of whether neutrophils initiate tissue damage and fibrosis or are recruited as a physiological response to tissue injury? The depletion of neutrophils and their enzymatic contents impacts bleomycin-induced pulmonary collagen deposition in some animal models [8–10]. However, results vary and translate poorly to the disease process in humans. The impact of neutrophils in the injured and fibrotic lung in patients with ILD remains unknown; do neutrophils amplify the fibrotic process, or is this a physiological attempt to repair the damaged pulmonary architecture?

The current review aims to 1) summarise the literature hypothesising the role of the neutrophil as a biomarker and contributor to the pathogenesis of ILD; 2) discuss gaps that warrant further investigation; and 3) consider novel, neutrophil-centric therapeutic options.

### Search methods

For this narrative review, retrospective and prospective studies (restricted to those in the English language) published from inception to June 2024 in peer-reviewed journals were identified by interrogation of PubMed. Search terms included “interstitial lung disease”, “idiopathic pulmonary fibrosis”, “IPF”, “neutrophils”, “polymorphonuclear cells”, “lung fibrosis”, “neutrophil extracellular traps”, “neutrophil elastase”, “proteinase” and “transforming growth factor beta”. References from relevant articles were also examined.

## IPF pathophysiology

### Fibrosis driven by alveolar epithelial damage and myofibroblasts

**FIGURE 1:**
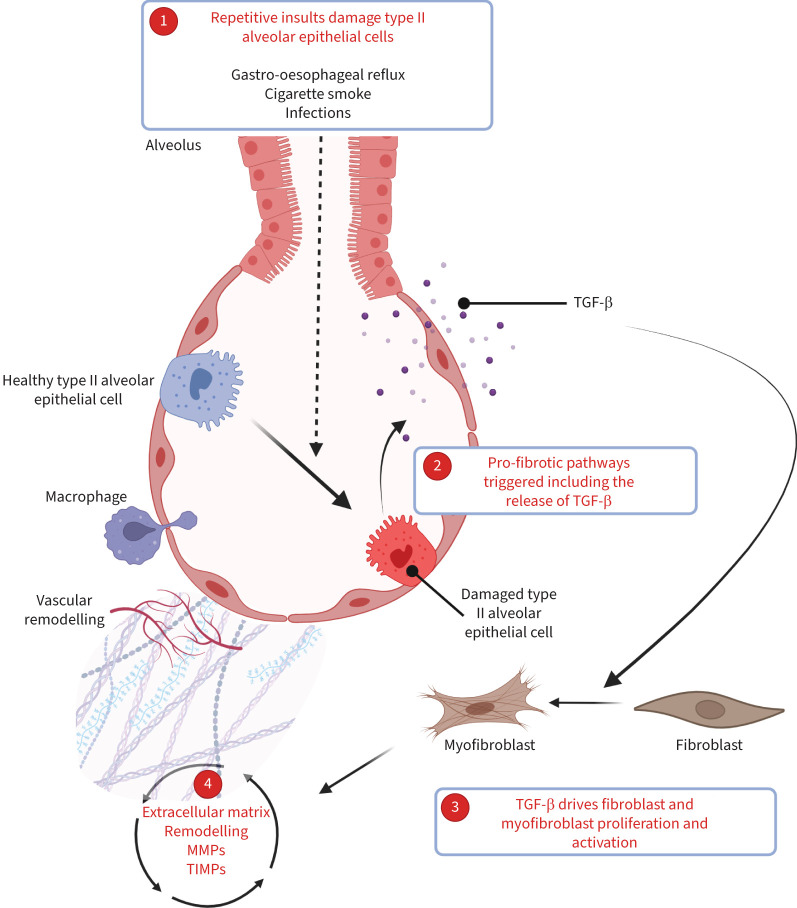
Outline of the pathophysiology of idiopathic pulmonary fibrosis. 1) Repetitive insults damage type II alveolar epithelial cells which undergo dysregulated apoptosis and repair [11], which triggers 2) pro-fibrotic pathways including release of the pro-fibrotic growth factor transforming growth factor (TGF)-β [12]. 3) TGF-β drives mesenchymal cell, particularly fibroblast and myofibroblast proliferation and activation [13]. 4) This results in a self-perpetuating cycle of remodelling, regulated by metalloproteinases (MMPs) and tissue inhibitors of metalloproteinases (TIMPs) and increasing deposition of extracellular matrix material [14]. The role of macrophages is becoming increasingly evident [15].

Common mechanisms are probably shared among ILDs. Although the aetiology of IPF is unknown, its pathogenesis is highly characterised. It is considered that repetitive cellular injury drives type II alveolar epithelial cell apoptosis, prompting a cascade of dysregulated lung tissue repair, remodelling and neo-angiogenesis (figure 1) [11]. An array of repetitive insults are thought to drive the initial damage including gastro-oesophageal reflux, cigarette smoke and infection [16]. Pro-fibrotic growth factors such as transforming growth factor (TGF)-β and alterations to the extracellular environment promote a positive feedback loop of fibroblast proliferation, myofibroblast transdifferentiation and dysregulated collagen turnover [11]. Lung injury and subsequent fibrogenesis in this context was thought to be related to epithelial–mesenchymal interactions with minimum involvement from the innate immune system. However, the importance of macrophages in this process is becoming increasingly evident. This includes fibroblast activation through the release of pro-fibrotic cytokines, such as TGF-β [15].

### The proposed role of TGF-β

TGF-β is the most studied pro-fibrotic growth factor and has three isoforms (TGF-β1, -β2 and -β3), with TGF-β1 considered the most relevant in ILD [17]. TGF-β1 is a ubiquitous molecule that is secreted and sequestered in the extracellular matrix as a large latent complex [18]. TGF-β bioavailability is largely regulated by the activation of TGF-β1 when it is released from the latent complex. An array of environmental changes, such as increased mechanical tension, have been reported to activate latent TGF-β1, all of which are related to changes in the extracellular matrix [18]. Immunohistochemistry of lung sections from patients with IPF has confirmed increased TGF-β1 expression in both early and advanced lung fibrosis [19]. Similarly, serum levels of TGF-β1 are raised approximately three-fold in patients with “idiopathic interstitial pneumonia” compared to healthy controls [20]. This also occurs in other ILD types with TGF-β1 levels significantly increased in patients with systemic sclerosis (SSc)- and rheumatoid arthritis (RA)-associated lung fibrosis [21, 22].

TGF-β signalling plays an important role in the lung epithelial cellular response to injury, promoting FasL-mediated apoptosis [23] and epithelial-to-mesenchymal transition [24]. Impaired regeneration of alveoli can promote elevated mechanical tension leading to further activation of the TGF-β signalling pathways in type II alveolar epithelial cells [12]. TGF-β can then drive collagen deposition and extracellular matrix remodelling through the activation of fibroblasts, into α-smooth muscle actin (SMA)-expressing myofibroblasts [13], with an imbalance between matrix metalloproteinases (MMPs) and their cognate inhibitors (tissue inhibitors of metalloproteinases (TIMPs)) [14] either increasing or reducing collagen degradation.

## Neutrophils and fibrotic lung tissue

Pathological studies have identified both peripheral and local neutrophilic abnormalities in ILDs [7, 25, 26], raising the possibility that this reflects either a reaction to the fibrotic process or that neutrophils have a direct role in disease progression.

### Neutrophil counts in BALF

Elevated neutrophil content in the BALF of ILD patients was first reported in the 1990s [25–27], followed by numerous studies. The clinical impact of these observations was subsequently described in the 2012 official ATS clinical practice guideline, where IPF is among the differential diagnoses of a BALF “neutrophil cellular pattern” [28]. Most studies have reported BALF neutrophil percentage rather than count, making it difficult to interpret the real change in neutrophil recruitment. One study noted that both absolute neutrophil count and lymphocyte count were significantly greater in IPF BALF than healthy controls [29], and another reported that both neutrophil percentage and total cell count were elevated in IPF patients [30]. Nevertheless, some studies have failed to confirm this, with IPF BALF neutrophil count similar to controls [31].

It is evident that a significant number of IPF patients have an elevated neutrophil percentage in the BALF. In IPF, 67% and 56% of patients were noted to have a BALF neutrophil percentage >3% [32] and >5% [33], respectively. In SSC-ILD, 47% of patients were noted to have a BALF neutrophil proportion of >4% [34]. These data suggest that neutrophil numbers are raised in the BALF of ILD patients, but it is important to consider that a reduction in other cell types could reflect this proportional increase. For example, some have suggested that BALF lymphocytosis is protective against fibrosis [33, 35].

Therefore, it is important to determine the clinical significance of elevated neutrophil percentage on disease progression. Several studies have reported an association between neutrophil content in BALF and the extent of segmental/lobar fibrosis and mortality [27, 36–38]. This includes the finding that in IPF patients, neutrophil counts as well as percentages were higher in BALF collected from lobes that were seen to have more extensive disease on imaging [37]. Elsewhere it has been found that with each doubling of neutrophil percentage in the BALF of IPF patients, there was a 30% increased risk of mortality [32]. Mortality was not associated with BALF eosinophil or lymphocyte percentage, suggesting the importance of neutrophils [32], although at variance with others [34]. The prognostic importance of neutrophils is likely not limited to IPF, with Watase *et al.* [38] finding that increased neutrophil percentage in BALF was also predictive of PF-ILD and subsequent mortality.

However, neutrophil activity may be more critical than their number. Increased neutrophil elastase (NE)–α_1_-antitrypsin (AAT) complexes have been observed in IPF patients, implying increased neutrophil degranulation [31]. Citrullinated histone 3, a marker of NET formation (a feature of neutrophil activation and also capable of tissue damage), is elevated in the BALF of patients with IPF [39]. However, incomplete demographic matching of IPF and control groups probably influences interpretation of these findings.

### Neutrophil counts in blood

Several studies have reported that circulating neutrophil counts are increased in patients with IPF and RA-ILD [40–42]. However, average counts are within the normal reference range. Careful consideration of the clinical significance of this potentially small, but statistically significant, increase from controls in ILD patients is needed to avoid over-interpretation. The lack of information concerning the spread of data makes the current literature difficult to interpret. For instance, an elevated neutrophil count defined by some as >7.5×10^9^ cells·L^−1^ reflects lung function decline/progression [43, 44], although the proportion of patients above this threshold is not described, and hence patient-specific impact is unknown.

Like in BALF, it may be that the relative contribution of neutrophils, rather than absolute count, is critical in ILD patients. It is important to determine this, as the former could merely reflect a reduction of other immune cells. There have been numerous studies of the association of neutrophil/lymphocyte ratio (NLR) [38]. A meta-analysis summarised that NLR was greater in ILD cohorts, compared to non-ILD participants, and associated with worse prognoses [45]. Many studies that identified NLR as a potential biomarker either did not comment on neutrophil count alone or reported no association with outcomes [46–48], suggesting that a reduction in lymphocyte count may have reflected the changes seen.

However, it is inappropriate to dismiss circulating neutrophil levels, as biomarkers in this disease process on the basis of ratio to lymphocytes alone. For example, Achaiah *et al.* [44] noted that a raised peripheral neutrophil count (defined as >7.5×10^9^ cells·L^−1^) in IPF patients was associated with mortality, whereas NLR was not. Furthermore, circulating neutrophil count has been noted in other studies to be associated with IPF development, severity and mortality [42–44, 49], and survival in RA-ILD [50]. A large prospective study may help answer the question of whether peripheral neutrophil count alone is a prognostic biomarker in ILD and together with paired BALF samples determine whether blood data reflects both BALF and tissue neutrophil counts and activity. Thus, interpreting blood data alone is complex, as this reflects neutrophil granulopoiesis, partitioning, extravasation into target organs and tissue clearance, and does not necessarily reflect the degree of local neutrophil recruitment and activation *in situ*.

Overall, the evidence supports the concept that neutrophils are an important part of the immune landscape of ILD and appear to be associated with fibrosis severity. Whether this is reflected solely by an increase in absolute number and/or a reduction in other immune cells is not clear. Clearly this needs further study. Presently the role of neutrophils needs greater clarification in the pathophysiology itself, but also any relationship to acute exacerbations of ILD, which are frequent and associated with significant morbidity and mortality [51]. For instance, in coronavirus disease 2019 (COVID-19), amplified systemic neutrophil counts, enzymes and NETs are associated with post-COVID-19 interstitial lung changes [52]. Whether the neutrophil response in acute exacerbations of ILD have such an effect is unknown.

### Neutrophil localisation within the fibrotic lung

The localisation of neutrophils in the lung parenchyma in ILD remains uncertain and there are no quantitative data of neutrophil infiltration in fibrotic lung. Usual interstitial pneumonia (UIP) that defines the histopathological and radiological pattern in IPF, and is present in many non-IPF PF-ILDs, is characterised by spatially and temporally heterogeneous fibrotic changes including reticulation, honeycombing and fibroblastic foci, with reported scarcity of ground-glass changes and inflammatory infiltrates including neutrophils [53, 54].

In contrast, neutrophilic features have been reported by others as “characteristic” of IPF, with neutrophils present in the lung interstitium, particularly dense fibrotic areas, and adjacent to areas of honeycombing, alveolar septa and endothelial cells in the pulmonary microvasculature [7, 31]. Degranulation marked by the presence of NE has been noted in IPF lungs, but not in control tissue [31]. This enzyme, alongside myeloperoxidase (MPO), another granule protein, has also been identified within the pulmonary vasculature of IPF lung tissue [55]. In addition, there is colocalisation of the markers of NETs, including citrullinated histones, MPO and extracellular DNA in fibrotic lung, but not in control tissue [55]. These studies challenge the didactic histopathological definition of UIP and suggest that neutrophils have the potential to increase inflammation and tissue destruction in these diseases. The temporal nature of neutrophil infiltration is unknown and may change with time, which could explain disparities with previous histopathological reports. Detailed histological examination of lung tissue at variable disease stages is required to clarify the relationship to disease trajectory.

## Are neutrophil-mediated repair mechanisms disrupted or dysfunctional?

**FIGURE 2:**
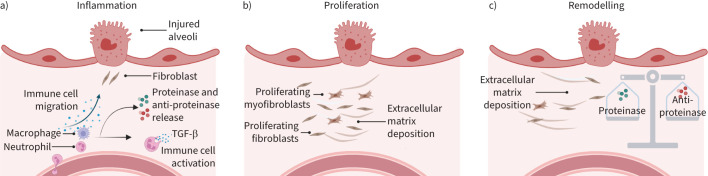
Stages of wound repair. Following tissue injury, immune cells, including neutrophils are recruited a) to remove pathogens/unwanted debris, and release cytokines, such as interleukin-8 and transforming growth factor (TGF)-β, and proteinase/anti-proteinases. b) Fibroblasts/myofibroblasts migrate, proliferate and deposit extracellular matrix material. c) Extracellular matrix material, particularly collagen, is remodelled resulting in increased tensile strength and organised fibrotic tissue. This process is dependent on a delicate balance of proteinases and anti-proteinases [56].

There is growing evidence that neutrophils play a ubiquitous role in tissue repair (figure 2). A murine two-hit (acid aspiration and mechanical ventilation) pulmonary injury model demonstrated that neutrophils rapidly migrate to damaged lung, with tissue remodelling and collagen deposition evident soon after [57]. The role of neutrophils in lung repair is further supported by murine ventilator-induced lung injury models where lungs from neutropenic mice demonstrate delayed repair [58]. This is also seen in thermal hepatic injury where neutrophils are crucial for the reduction of injury size, collagen deposition and revascularisation [6]. Dermal wound closure models investigating the effect of deficiencies in the chemotactic receptors formyl peptide receptor 1 and 2 have also demonstrated that early neutrophil recruitment is critical for normal wound healing [59].

Neutrophil dysfunction has been noted in ageing [60]. The importance of neutrophil-mediated repair mechanisms may therefore become more apparent with increasing age, as evidenced by neutrophil depletion markedly delaying wound healing in aged mice [61]. This is relevant, as many ILDs, such as IPF, occur more commonly in older adults.

Mechanisms of neutrophil-driven repair mechanisms are currently unclear. Neutrophil-derived proteinases, such as MMP-9 [58] appear important in lung injury repair in animal models. Alveolar epithelial damage is considered an early step in IPF pathogenesis. Activation of β-catenin signalling through NE-mediated cleavage of E-cadherin is a proposed mechanism by which neutrophils regulate alveolar epithelial repair [62]. Neutrophil depletion impairs type II alveolar epithelial cell proliferation [63]. It is unknown whether neutrophil-mediated repair mechanisms such as these are functional or dysregulated in patients with ILD.

Pro-resolution effects can also be detrimental. A further murine model demonstrated that neutrophilic inflammation at the saccular stage of lung development can result in aberrant elastic fibre generation, probably through inhibitory effects of NE on elastic fibre assembly [64]. Therefore, neutrophil repair processes may, in part, “plug the damage”, but with abnormal extracellular matrix material and structure. This needs to be explored in ILDs, as any disruption to the delicate alveolar–capillary interface required for efficient gas exchange could have major clinical effects.

## Potential impact of neutrophils in the fibrotic microenvironment?

Experimental work to date investigating the impact of neutrophils in the pulmonary fibrotic microenvironment have been limited to animal models and simple *in vitro* cell culture systems.

### Animal models

The few animal models used to investigate lung fibrosis pathophysiology poorly reflect progressive pulmonary fibrosis in humans. The bleomycin model is the most established, as it develops some of the histological hallmarks seen in IPF, is reproducible and readily accessible [65]. However, it is far from ideal as it displays a degree of reversibility, unlike human disease, and is associated with a markedly greater inflammatory response [65].

Table 1 summarises animal models that have been performed to investigate the effect of neutrophil manipulation on lung fibrosis. Neutrophils are recruited to the BALF within 24 h of bleomycin administration in murine models [66]. Neutrophils remain elevated alongside increased NE activity even at day 14 [10]. The effect of this neutrophil recruitment is unclear, with neutrophil depletion either having a negligible effect or increasing collagen content within 1–2 weeks [8, 9, 66, 67]. This suggests that neutrophils may be protective or amplify bleomycin damage. However, inhibition of NE consistently reduces lung collagen content in bleomycin models, suggesting the enzyme is a potential driver of fibrosis [10, 68, 69].

**TABLE 1:** Animal models investigating the effects of neutrophils and related contents on lung fibrosis

First author, year [reference]	Fibrosis model	Type of neutrophil manipulation	Impact on lung collagen content	Other effects
**Neutrophil depletion**				
Thrall, 1981 [8]	Bleomycin rat model	Neutrophil depletion: anti-neutrophil serum	Increased at 1 week No effect at 1 month (neutrophil count restored at week 1)	
Clark, 1982 [9]	Bleomycin hamster model	Neutrophil depletion: anti-neutrophil serum	Increased at days 8 and 12	
Manoury, 2007 [66]	Bleomycin mouse model	Neutrophil depletion: anti-neutrophil antibody	No effect at day 14	Reduced pro-MMP-9 activity at day 1
Lv, 2017 [67]	Bleomycin mouse model	Neutrophil depletion: anti-mouse Ly6G mAb	No effect at day 21	
**Neutrophil elastase**				
Chua*,* 2007 [68]	Bleomycin mouse model	NE-null mice	Reduced at day 30	Reduced active TGF-β1 levels in lung tissue at day 7
Takemasa, 2012 [10]	Bleomycin mouse model	NE inhibitor (sivelestat)	Reduced at days 7 and 14	Sivelestat reduced bleomycin-induced increase in active TGF-β1 levels and phosopho-Smad2 in lung homogenates
Gregory, 2015 [69]	Asbestos mouse model	NE-null mice Small-molecule inhibitor of elastase	Reduced at day 14 Reduced at day 14	Reduced fibroblasts and myofibroblasts
Cheng, 2019 [70]	Bleomycin +/− particulate matter mouse model	NE inhibitor (sivelestat)	Reduced at day 14	Sivelestat reduced bleomycin-induced phosphorylation of Smad2/Smad3 and α-SMA
**NET manipulation**				
Suzuki, 2020 [71]	Bleomycin mouse model	PAD inhibitor (Cl-amidine) PAD4 knockout mice	Reduced at day 21 in PAD4 knockout mice	Cl-amidine reduced bleomycin-induced NET formation in the blood, alveolar and interstitial spaces Haematopoietic cell grafts from PAD4 knockout mice reduced bleomycin-induced lung fibrosis
Riehl, 2023 [39]	Bleomycin mouse model	Neutralising monoclonal anti-histone H2A/H4 antibodies	Reduced at 4 weeks	TGF-β1 reduced in BALF at weeks 2 and 4

NET: neutrophil extracellular trap; MMP: matrix metalloproteinase; Ly6G: lymphocyte antigen 6G; mAb: monoclonal antibody; NE: neutrophil elastase; TGF: transforming growth factor; SMA: smooth muscle actin; PAD: pan-peptidyl arginine deaminase; BALF: bronchoalveolar lavage fluid.

To understand the role of the neutrophil in disease it is important to characterise the recruitment process, cell maturity and activation status both peripherally and within the lung microenvironment, as this will influence the presence and degree of effector functions. This information is currently lacking in patients with ILD.

A sustained increase in “aged” (CXCR4-high and CD62L-low) neutrophils in the blood and BALF of bleomycin-treated mice has been noted [72]. Neutrophil activation markers such as NE and MPO were elevated in the BALF of bleomycin-treated mice [72]. The same study suggested that tissue-resident macrophages may be central to conducting neutrophil recruitment to the alveolar spaces in lung fibrosis [72]. With the described limitations of animal models, it is important to determine the state and associated effector functions of neutrophils from patients with ILDs, not only to determine any concordance with animal models, but to identify modulation pathways.

### Proteinases

Neutrophils contain at least four types of granules, storing serine proteinases including NE, proteinase 3, cathepsins G and S and MMPs, such as MMP-8 (neutrophil collagenase) and MMP-9 (gelatinase-B). These are all released extracellularly during recruitment and activation and especially during cell death, leading to NET formation [73]. Whether these functions differ in patients with lung fibrosis is unknown and important to determine as homeostasis of extracellular matrix deposition and degradation is probably determined by the balance with their inhibitors (TIMPs and AAT/secretory leucocyte protease inhibitor) in the lung [74].

**FIGURE 3:**
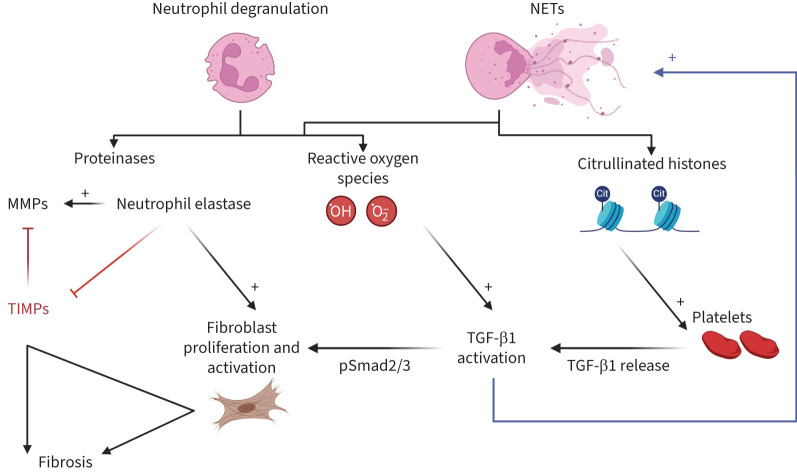
Potential mechanisms by which neutrophils drive lung fibrosis. Upon neutrophil activation (degranulation and/or neutrophil extracellular trap (NET) formation) there may be release of 1) proteinases, such as neutrophil elastase and matrix metalloproteinases (MMPs); 2) reactive oxygen species; and 3) citrullinated (cit) histones. All these substances may promote fibrosis through altering the balance of MMPs/tissue inhibitors of metalloproteinases (TIMPs), driving fibroblast proliferation and activation, and increasing the bioavailability of transforming growth factor (TGF)-β1. Active TGF-β1 may perpetuate this process by driving NET formation. ^.^O_2_^−^: superoxide anion; ^.^OH: hydroxyl free radical; pSmad2/3: phosphorylated Smad2/3.

NE is the most characterised neutrophil derived proteinase in ILD with increasing evidence that it is important in fibrosis development. NE levels are elevated in IPF BALF [75]. Animal and *in vitro* studies have demonstrated the importance of NE in models of lung fibrosis, highlighting it as a potential therapeutic target. NE-null mice are protected from asbestos- and bleomycin-induced lung fibrosis [68, 69]. NE promotes lung fibroblast proliferation and myofibroblast transdifferentiation *in vitro*, the latter appearing to be independent of TGF-β and phosphoinositide-3-kinase, but dependent on Smad signalling [69]. Furthermore, NE inhibition by sivelestat reduces bleomycin-induced pulmonary fibrosis and neutrophilic inflammation [10]. Finally, NE has also been noted to induce collagen deposition in a bleomycin model perpetuated by particulate matter exposure, through activation of the Smad2/Smad3/α-SMA pathway [70]. NE can itself impact the MMP–TIMP balance by inactivating TIMP [76] and activating MMPs, which is worthy of further study as a mechanism in ILD. Figure 3 summarises the proposed mechanisms by which neutrophils, including proteinases, may drive fibrosis.

Neutrophil release of other proteinases may also contribute to tissue and vascular remodelling in the lung. MMP dysregulation is thought to play a critical role in alveolar permeability and deranged collagen turnover, with MMP-7 silencing significantly attenuating fibrosis in bleomycin rat models [77]. In response to tissue injury, the best *in vivo* evidence shows that MMP-7 drives re-epithelialisation and neutrophil influx [78]. This may be helpful in an acute setting, but a prolonged/excessive response, for example in the context of fibrosis, can also be damaging [78]. Neutrophils have been reported as an important cellular source of MMP-9 in lung inflammation [79]. MMP-8 and -9 are elevated in the BALF of IPF patients, especially in those with rapid lung function decline, and MMP-8 relates to neutrophil count and BALF collagenolytic activity [74, 80]. MMP-8 knockout mice treated with bleomycin develop less lung fibrosis (despite greater lung inflammation) than wild-type mice [81]. However, MMP-9 knockout had little effect in an experimental lung fibrosis model [82], suggesting that the former was more important in this process.

Therefore, targeting proteinases as a potential therapeutic strategy in IPF seems logical, but potentially at risk of offsetting their regulatory roles. Clearly a better understanding of the potential roles and interaction of these individual enzymes and their relationship to neutrophil inflammation and fibrosis is essential in the development of such specific mediator therapy.

### TGF-β activation

As indicated earlier, TGF-β is generally accepted as a central mediator of the fibrotic process and may be modulated by neutrophils by the following methods.

1) NE can activate TGF-β1 in the extracellular matrix while silvestat (a specific NE inhibitor) reduces active TGF-β1 levels and its downstream signalling molecule phospho-Smad2, but not total TGF-β1 [10]. NE-mediated downstream signalling molecules have been demonstrated in bleomycin animal models [68, 70]. NE is also able to release latent TGF-β1 from epithelial and endothelial cell matrices *in vitro*, but is unable to activate latent TGF-β1 in this setting [83]. NE directly activates MMPs, which themselves activate TGF-β1, although this interplay in the fibrotic lung remains to be confirmed [84, 85].
2) Neutrophils may contribute to TGF-β bioavailability *via* alternative pathways. The bleomycin murine model suggested that externalised histones released on NET formation may promote TGF-β1 release from platelets [39]. Peripheral blood neutrophils themselves contain and secrete active TGF-β1 in response to phorbol 12-myristate 13-acetate stimulation [86]. Asbestos-derived ROS have been shown to activate latent TGF-β1 in A549 cells [87], although it is unknown whether neutrophil-generated ROS also increases TGF-β1 activation, especially in the lung. Oxidant-driven activation of TGF-β has been demonstrated in fibroblasts, wherein cellular influx of superoxide through chloride channels caused extracellular signal regulated kinases 1/2 and p38 mitogen-activated protein (MAP) kinase dependent activation and subsequent upregulation of TGF-β1 [88]. By this rationale, activated neutrophils, which produce superoxide as a primary microbe-killing mechanism, are capable and perhaps likely to induce the same processes of oxidant-driven TGF-β1 activation as seen in fibroblasts.

### Neutrophil extracellular traps

NETs are web-like DNA- and protein- (such as proteinases) containing structures, that can be rapidly released upon neutrophil stimulation and especially cell death. NETs have been identified in IPF lung tissue and are increased in ILD BALF [55]. The key determinants of NET formation are unknown here, although TGF-β may be important [89]. Primary human lung fibroblasts stimulated with NETs drive myofibroblast differentiation, collagen production and promote alveolar epithelial cell damage through histone release [90, 91]. However, the mechanism by which NETs influence other changes in pulmonary epithelial and mesenchymal cells remains unclear. Again, persistence of neutrophils and NETs in the lung of IPF patients may enhance and perpetuate the fibrosis element and/or impair wound healing [92].

### Barriers to neutrophil research in ILDs

Neutrophils are vulnerable to activation and degranulation *ex vivo* and these features are heavily influenced by processing time and neutrophil isolation methods [93]. This fragility is likely a significant reason for our relatively poor understanding of neutrophils contribution to lung fibrosis including lack of single cell ribonucleic acid-sequencing/proteomic data, which could provide important insights.

## What drives neutrophils to the lung in ILD?

### Alveolar epithelial cell damage?

**FIGURE 4:**
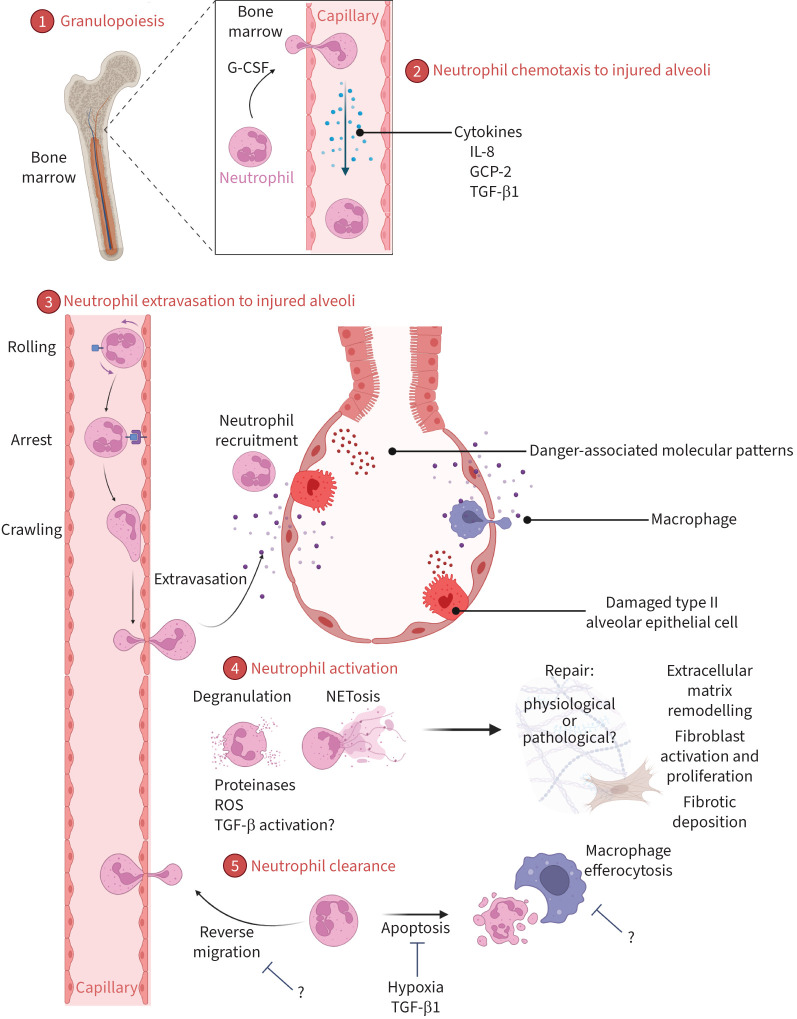
Proposed pathway of neutrophil response to injured alveoli in interstitial lung diseases. 1) Neutrophils are produced by the bone marrow in response to factors such as granulocyte colony-stimulating factor (G-CSF) [94, 95], and 2) migrate to the site of alveoli injury down a chemotactic gradient. 3) Neutrophils extravasate from the pulmonary capillaries into the alveoli interstitium [96] in response to chemokines and danger signalling molecules released from damaged type II alveolar epithelial cells [16, 97, 98]. 4) On arrival, neutrophils are activated, degranulating and/or undergoing neutrophil extracellular trap formation (NETosis) [55], which releases enzymes, such as proteinases, transforming growth factor (TGF)-β1 and reactive oxygen species (ROS). These mediators attempt to drive repair processes including extracellular matrix remodelling. This may activate fibroblasts and result in fibrotic deposition [69, 91]. 5) Following activation, neutrophil clearance through reverse transmigration out of the lungs and/or apoptosis is required to maintain homeostasis. Factors such as hypoxia [99] may reduce the homeostatic clearance of senescent neutrophils. IL: interleukin; GCP: granulocyte chemotactic protein.

Neutrophil mobilisation has been suggested as a physiological response to the type II alveolar epithelial cell damage that occurs early in the pathogenesis of IPF. Figure 4 summarises the potential stages of neutrophil migration to and from damaged alveoli in ILDs. Damaged epithelial cells release danger-associated molecular patterns (DAMPs) that act on pattern recognition receptors (PRRs) in order to drive an immune and potentially reparative response [97]. Expression of these danger signals may account for migration of neutrophils to fibrotic lungs in close proximity to areas of alveolar damage in IPF [98]. This danger signalling may be an essential component of the physiological pro-healing response [59].

However, once in the lung, the neutrophil driven “remodelling” may also be detrimental to the interstitial architecture. For example, the PRR, formyl peptide receptor 1, heavily expressed on neutrophils, and its ligands, appear to be critical in bleomycin-induced neutrophil recruitment and subsequent fibrosis in the lung, but not in the kidney or liver [97]. Neutrophils themselves may drive DAMP release, thereby amplifying and perpetuating neutrophil infiltration, through proteinase mediated extracellular matrix degradation products, such as fibronectin, having the propensity to activate toll-like receptors [100], although this has yet to be confirmed in the lung.

### Increased granulopoiesis?

Granulocyte colony-stimulating factor (G-CSF) is a major regulator of both differentiation of myeloid progenitors into mature neutrophils and their release from the bone marrow (figure 4) [94]. Greater G-CSF levels have been noted in IPF BALF than in health [101]. BALF G-CSF levels were related to the BALF neutrophil count and negatively associated with disease severity as determined by forced vital capacity (FVC) [101]. Neutrophil chemotaxis was increased towards BALF derived from IPF patients, and was abrogated by G-CSF neutralisation, indicating that G-CSF is probably an important chemoattractant in this condition [101]. G-CSF exacerbates lung injury in bleomycin rat models through a neutrophil-dependent mechanism [95]. Such a mechanism suggests a process whereby neutrophil production and recruitment to the damaged lung by G-CSF is increased, potentially exacerbating the tissue damage. However, G-CSF may also act as an antifibrotic by promoting bone marrow mesenchymal stem cell mobilisation to injured tissue [102]. Whether this is important in human disease development and how it alters neutrophil phenotype, recruitment and activation are clearly worth further study.

### Altered neutrophil migration and chemotaxis?

The chemokine and endothelial environment changes in response to bleomycin in animal models increasing the release of 1) chemoattractants interleukin (IL)-8 and monocyte chemoattractant protein-1, and 2) upregulating expression of adhesion molecules such as E-selectin, intracellular adhesion molecule (ICAM)-1 and vascular adhesion molecule 1 by pulmonary microvascular endothelial cells [96]. Using an *in vitro* flow system, neutrophil adhesion to human umbilical vein endothelial monolayers was increased by bleomycin treatment, independent of ICAM-1, CD18 (a β2 integrin), and E-selectin [96]. Alveolar/capillary integrity may also be influenced by changes in the fibrotic microenvironment, such as hypoxia which increases neutrophil adhesion, transendothelial migration and expression of α_M_β_2_ and α_X_β_2_ integrins *in vitro* [55].

Although these chemokine and endothelial changes have been seen in animal models and *in vitro*, the pertinent mediators in human ILDs are less clear. There is evidence that the chemokine milieu in BALF is dysregulated increasing neutrophil chemotactic response in IPF compared to health [30]. IL-8 is one of the major chemoattractants generated in the lung, acting *via* the neutrophil cell surface receptors CXCR1 and CXCR2. One study demonstrated significantly greater IL-8 concentrations in the supernatant from cultured IPF-derived endothelial progenitor cells than control cells [103]. Greater neutrophil transwell migration was noted *ex vivo* towards the IPF conditioned media than control and was abolished by an anti-IL-8 antibody [103]. Furthermore, IL-8 staining was noted in the vessels of IPF lung tissue, but not control [103]. IL-8 levels in the serum and BALF are significantly higher in patients with ILD and serum IL-8 levels relate to the percentage of neutrophils in the BALF, supporting its role in neutrophil mobilisation [104, 105]. In addition, serum IL-8 levels are negatively related to lung function indicators of disease severity, FVC and gas transfer, arterial oxygen tension and survival [104, 106]. These data suggest that IL-8 is a marker of the severity and progression of IPF possibly reflecting neutrophil recruitment and activation.

Other chemokines may be important in neutrophil mobilisation in ILDs. The chemokine granulocyte chemotactic protein 2 is upregulated in the BALF of IPF patients and bleomycin-challenged mice [107]. Furthermore, neutralisation of granulocyte chemotactic protein 2 reduces early inflammation, neutrophil influx, TIMP-1 expression and fibrosis in bleomycin murine models, suggesting that the inflammatory activity of this cytokine in this milieu probably has a role [107].

### Amplified/abnormal cell recruitment (TGF-β1)?

The generation of classical neutrophil chemoattractants may be central to the physiological neutrophil response in IPF. However, in the complex inflammatory milieu it remains possible that cytokines not conventionally recognised as chemoattractants may prime neutrophil responses or the destructive processes in the lung matrix may amplify the neutrophil response.

TGF-β1 (a key cytokine in the fibrotic response) can also act as a neutrophil chemoattractant through binding to high-affinity TGF-β1 receptors present on the neutrophil surface [108, 109]. The chemoattractant capabilities of TGF-β1 was first demonstrated both *in vivo* following intra-articular TGF-β1 injection in rats and *in vitro* on isolated human neutrophils [86]. TGF-β1 may affect neutrophil chemotaxis through redistribution and polymerisation of actin filaments *via* the p38 MAP kinase pathway [110]. Alternatively, it may occur *via* fibronectin–VLA-5 integrin binding on the neutrophil surface [111].

TGF-β acting as an additional chemoattractant in the fibrotic microenvironment is also plausible. However, TGF-β1 inhibition does not attenuate the chemotactic effects of supernatant derived from human lung fibroblasts that had been treated with bleomycin [112], whereas anti-IL-8 and anti-G-CSF antibodies do reduce this response [112], suggesting minimal TGF-β1 contribution to the overall effect. In addition, others noted that TGF-β1 had no chemotactic activity *in vitro*, providing further confirmation of a lack of effect on this process [113, 114]. However, TGF-β1 has been shown elsewhere to inhibit IL-8 dependent neutrophil migration through endothelial cells *in vitro* by downregulating endothelial cell expression of the adhesion molecule, E-selectin [115].

Therefore, this key fibrotic cytokine may influence overall neutrophil recruitment in IPF either directly or indirectly, but further studies are required to clarify the mechanism and explore its contribution in neutrophil influx in ILDs and the relationship to progression and severity.

### Impaired neutrophil clearance/resolution?

Following recruitment as part of a physiological response to injury, neutrophils need to undergo clearance as persistence may enhance fibrosis, tissue damage and inflammation in the pulmonary interstitium as in models of lung fibrosis and fibroproliferative acute respiratory distress syndrome [116–118].

Macrophage efferocytosis is a major mechanism of neutrophil clearance in the lung as part of the pro-resolution response. Efferocytosis of apoptotic alveolar cells is impaired in alveolar macrophages isolated from IPF patients [119]. It is not known whether there is a similar defect in the efferocytosis of senescent neutrophils. Although TGF-β1 has been noted to increase macrophage mediated clearance of apoptotic neutrophils *in vitro*, its effects on ILD-derived cells are unknown [120].

Pro-survival signals in the fibrotic microenvironment may contribute to prolonged residence and activity of neutrophils in lung tissue. These may include TGF-β and hypoxia (with subsequent activation of the hypoxia-inducible factor 1α hydroxylase oxygen-sensing pathway) increasing neutrophil longevity [99, 121]. In addition, increased neutrophil retention upon priming [122] could be potentially damaging to the tissues.

The complex nature of neutrophil mobilisation and clearance has only been covered briefly here with tissue injury likely to be the primary trigger. The persistent neutrophilia is recognised, but the mechanisms by which this carefully controlled process of recruitment and removal apply or are altered in ILDs are relatively unexplored. These include the impact of persistent neutrophilia on the fibrotic microenvironment beyond any physiological effect. Does the fibrotic milieu drive neutrophils to transition into a more damaging rather than reparative phenotype?

## Therapeutic potential in neutrophil modulation

Anti-inflammatory and immunomodulatory treatments continue to be the mainstay pharmacological therapies for ILDs that demonstrate significant inflammatory radiological changes. This was previously true for IPF until a landmark combination trial, of azathioprine, *N*-acetylcysteine and prednisolone was prematurely discontinued following interim results revealing increased mortality and hospitalisation in the intervention arm (www.ClinicalTrials.gov identifier NCT00650091) [123].

Currently, the only approved pharmacological therapies for IPF are the two antifibrotic agents, nintedanib and pirfenidone. Nintedanib is an antivascular endothelial growth factor receptor-2, fibroblast growth factor receptor-1 and platelet-derived growth factor receptor-α, -β multityrosine kinase inhibitor, having multifactorial effects *in vitro* including suppressing fibroblast proliferation and activation [124] and has now been extended to non-IPF PF-ILDs/PPF. Conversely, the precise mechanism of action of pirfenidone is unclear. Both drugs show signs of immunomodulatory effects, for example dampening bleomycin-induced neutrophil infiltration and fibrosis in animal models [125, 126], but a direct antineutrophil effect is not clear. With current therapeutic strategies only slowing disease decline, targeting alternative pathways, such as neutrophil effector functions, may provide a potentially important unmet need.

Failed attempts to safely antagonise TGF-β1 is an example of how problematic it can be to generate a therapeutic strategy against a target that has multifunctional homoeostatic effects. The same concept is true with an antineutrophilic strategy, where careful consideration is needed of potential adverse reactions that may occur with neutrophil modulation. The most notable risk is the increased susceptibility of serious recurrent infections as seen in patients with neutropenia. Neutrophil manipulation would need to be limited to clear pro-fibrotic pathways without adversely suppressing granulopoiesis, physiological recruitment and effector functions to minimise off-target effects. Supplementary table S1 describes potential strategies of neutrophil manipulation in ILD. However, more research is required to advance our understanding of the potential role that neutrophils have in ILD pathobiology to support the case for therapeutic targets.

## Conclusions

This review has highlighted the numerous plausible mechanisms by which neutrophils may play a role in the pathophysiology of lung fibrosis. The body of evidence appears sufficient to support neutrophils being present not solely as a reaction to the alveolar epithelial injury. However, their impact, whether reparative or pathological, on the delicate lung architecture has yet to be clarified. It is critical to understand the role as manipulation of neutrophil recruitment and function could provide a novel and much needed therapeutic strategy for patients with fibrosing ILDs but without physiological impairment of function.

**Questions for future research**

- Future research projects need to characterise in detail the phenotype and function of neutrophils residing in the circulation and pulmonary compartments of patients with ILD to identify any dysregulated pathways that contribute to fibrogenesis.
- Spatial multi-omic studies would provide detailed insights into neutrophil subpopulations including localisation and cell–cell interactions in fibrotic lung tissue.
- Focus on determining the role of neutrophils throughout the disease course and determining protective/pathological mechanisms is critical to identifying viable therapeutic targets.

## Supplementary material

10.1183/16000617.0139-2024.Supp1 **Please note:** supplementary material is not edited by the Editorial Office, and is uploaded as it has been supplied by the author.
Supplementary material ERR-0139-2024.SUPPLEMENT
