# Supplementary material for: Neutrophil dynamics in pulmonary fibrosis: pathophysiological and therapeutic perspectives
Source: Eur Respir Rev. 2024 Nov 27;33(174):240139. doi: 10.1183/16000617.0139-2024 (PMC11600124; doi:10.1183/16000617.0139-2024)
Supplement: Supplementary file 1 [file ERR-0139-2024.SUPPLEMENT.pdf]

| Targets | Drugs | Animal models/ Clinical trials |
|---|---|---|
| Neutrophil proteinase activation | Brensocatib (Dipeptidyl peptidase1 inhibitor) | Phase III clinical trial (STOP-COVID19 ISRCTN30564012 [1]) – brensocatib was not beneficial in the acute management of patients hospitalised with COVID-19. Brensocatib did not increase the rate of infection.<br><br>Phase II clinical trial (WILLOW: www.ClinicalTrials.gov identifier NCT03218917 [2]) - brensocatib extended time to first exacerbation in bronchiectasis patients.<br><br>Phase III clinical trial (ASPEN: www.ClinicalTrials.gov identifier NCT04594369) - results awaited. |
| Neutrophil elastase | Sivelestat (Neutrophil elastase inhibitor) | Bleomycin animal models – sivelestat reduced pulmonary fibrosis development [3,4].<br><br>Phase IV clinical trial in acute lung injury associated with systemic inflammatory response syndrome patients [5] - reasonable safety profile. |
| NET formation, such as peptidyl arginine deaminase (PAD) | PAD-4 inhibitor (JBI-589 - not yet trialed in humans) | Bleomycin murine models – PAD-4 knock out mice generated less NETs and lung fibrosis in response to bleomycin instillation than wild type mice [6].<br><br>JBI-589 blocked NET formation in an animal arthritis model [7]. |
| Granulocyte-macrophage colony stimulating factor | Namilumab (granulocyte-macrophage colony stimulating factor inhibitor) | Phase II clinical trial (CATALYST ISRCTN40580903 [8]) - namilumab reduced c-reactive protein levels in COVID-19 patients. Namilumab increased the rate of infection. |

**Supplementary Table 1 – Potential therapeutic strategies of neutrophil manipulation in interstitial lung diseases.**

Targets/ drugs that have shown promise in animal models and/ or clinical trials at manipulating neutrophil responses. These drugs have yet to be tested in patients with interstitial lung disease.

**REFERENCES**


1 Keir HR, Long MB, Abo-Leyah H *et al.* Dipeptidyl peptidase-1 inhibition in patients hospitalised with COVID-19: a multicentre, double-blind, randomised, parallel-group, placebo-controlled trial. *Lancet Respir Med* 2022; **10**: 1119.

2 Chalmers JD, Haworth CS, Metersky ML *et al.* Phase 2 Trial of the DPP-1 Inhibitor Brensocatib in Bronchiectasis. *N Engl J Med* 2020; **383**: 2127–2137.

3 Takemasa A, Ishii Y, Fukuda T. A neutrophil elastase inhibitor prevents bleomycin-induced pulmonary fibrosis in mice. *Eur Respir J* 2012; **40**: 1475–1482.

4 Cheng IY, Liu CC, Lin JH *et al.* Particulate Matter Increases the Severity of Bleomycin-Induced Pulmonary Fibrosis through KC-Mediated Neutrophil Chemotaxis. *Int J Mol Sci* 2019; **21**. doi:10.3390/IJMS21010227.

5 Aikawa N, Ishizaka A, Hirasawa H *et al.* Reevaluation of the efficacy and safety of the neutrophil elastase inhibitor, Sivelestat, for the treatment of acute lung injury associated with systemic inflammatory response syndrome; a phase IV study. *Pulm Pharmacol Ther* 2011; **24**: 549–554.

6 Suzuki M, Ikari J, Anazawa R *et al.* PAD4 Deficiency improves bleomycin-induced neutrophil extracellular traps and fibrosis in mouse lung. *Am J Respir Cell Mol Biol* 2020; **63**: 806–818.

7 Gajendran C, Fukui S, Sadhu NM *et al.* Alleviation of arthritis through prevention of neutrophil extracellular traps by an orally available inhibitor of protein arginine deiminase 4. *Sci Rep* 2023; **13**: 1–14.

8 Fisher BA, Veenith T, Slade D *et al.* Namilumab or infliximab compared with standard of care in hospitalised patients with COVID-19 (CATALYST): a randomised, multicentre, multi-arm, multistage, open-label, adaptive, phase 2, proof-of-concept trial. *Lancet Respir Med* 2022; **10**: 255.
